# Left Renal Artery Chronic Occlusion in the Setting of Extensive Intraprosthetic Mural Thrombus in an Abdominal Aortic Endograft

**DOI:** 10.7759/cureus.59624

**Published:** 2024-05-04

**Authors:** Javier B Chambi-Torres, Saleha Ozair, Larri Rudman, Sabas Gomez, George Michel

**Affiliations:** 1 Internal Medicine, Larkin Community Hospital, South Miami, USA; 2 Cardiology, Larkin Community Hospital, South Miami, USA

**Keywords:** thrombosis, aorta, renal artery, endovascular repair, abdominal aortic aneurysm

## Abstract

Thrombotic deposits within aortic endograft post-endovascular aortic aneurysm repair (EVAR) is a fairly well-recognized complication, yet their clinical significance remains inadequately understood. We present a rare case of extensive mural thrombus formation in an oncologic patient with a history of EVAR, emphasizing the importance of lifelong surveillance in abdominal aortic aneurysm (AAA)-EVAR patients. A 75-year-old female was admitted with refractory hypertension secondary to a medium-sized AAA, which exhibited an extensive mural thrombus, contributing to atrophic changes in the left kidney and likely chronic occlusion of the left renal artery. Factors contributing to thrombus formation generally include endograft configuration, aneurysm diameter, and patient-specific characteristics, such as a pro-thrombotic status conferred by metastatic lung cancer. This case underscores the necessity for comprehensive surveillance strategies post-EVAR. Recommendations advocate for a 30-day follow-up and lifelong annual surveillance, employing modalities such as color duplex ultrasound for detection of endoleaks and sac enlargement, with selective use of CT imaging. This case underscores the importance of continued vigilance and surveillance in patients undergoing EVAR, particularly those with complex medical histories, to mitigate potential long-term complications and optimize patient outcomes.

## Introduction

Thrombotic deposits forming within aortic endografts have been noted in both thoracic and abdominal regions. Despite being identified early in the era of endovascular aortic aneurysm repair (EVAR) techniques, there remains a paucity of data regarding their clinical relevance and implications [1]. EVAR has 20% to 30% more complications than open surgical repair, such as temporary renal impairment, graft-related endoleaks, device occlusion, migration, distal embolization, or femoral access site lesions [2]. Another less-reported complication is the intraprosthetic mural thrombus (IPMT) which can occur in one-fifth of post-EVAR cases [3]. In a study conducted by Oliveira et al., the occurrence of mural thrombus following EVAR was noted to be a prevalent event, occurring in 16.4% of the 473 EVAR cases examined [2]. The main concern about the formation of mural thrombus is that it could lead to occlusion or distal embolization, causing an ischemic leg or occlude circulation to vital organs [2]. We present an oncologic patient with an extensive abdominal aortic endograft mural thrombus who was found to have an atrophic left kidney and likely chronically occluded left renal artery to present this rare complication and highlight the importance of life-long surveillance in AAA-EVAR patients. A concise overview of the literature concerning the occurrence and clinical ramifications of this complication is also provided.

## Case presentation

We present a 75-year-old female who was admitted to ICU for better control of her refractory high blood pressure of 200/140 mmHg in the setting of a 7.1x5.8 cm abdominal aortic aneurysm (AAA). Upon admission, heightened concern for the patient's uncontrolled hypertension prompted an immediate abdominal CT angiogram, revealing an extensive mural thrombus.

The patient's past medical history was relevant for coronary artery bypass graft in 2006, AAA EVAR in 2011, hypertension, and metastatic lung cancer status post chemotherapy and radiation in 2020. On admission, the patient was oriented in person, time, and place, her heart rate was 59 beats per minute, and her blood pressure had decreased to 168/68 mmHg because of the 20 mg of nifedipine that was given to her in the emergency department. The cardiac evaluation revealed a regular rhythm with no identified murmurs, apical impulses, or jugular venous distension. Her laboratory showed mild hypokalemia (3.4 mmol/L), which was corrected and monitored, and mildly elevated troponin level (0.04), which was monitored by the Cardiology team.

Once in the ICU, blood pressure was controlled with different antihypertensives, and the patient was not a surgical candidate now unless she was transferred to a higher-capability center.

**Figure 1:**
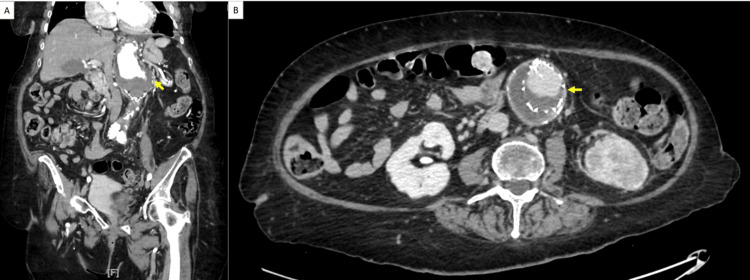
Endograft mural thrombus. (A) Coronal view (yellow arrow) and (B) axial view (yellow arrow).

**Figure 2:**
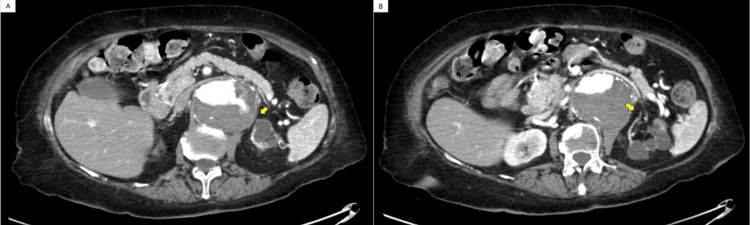
(A) Interruption of the main flow through the left renal artery (yellow arrow) and (B) small-caliber vessels are seen coursing towards the left kidney (yellow arrow).

Abdominal CT angiogram showed an extensive mural thrombus throughout the entire length of the AAA with the largest caliber at the renal level measuring approximately 7.1x5.8 cm without evidence of an endoleak or rupture at the level of the abdominal aortic stent and kissing iliac stents (Figure 1). At the site, which looked like the left renal artery, which was not well visualized, an area of contrast infiltrated within the thrombus. Her left kidney appeared to be atrophic/cystic in appearance at the superior pole and interpolar regions (Figure 2).

Finally, the palliative care service spoke with the patient and her family, and the acceptance of hospice care was taken.

## Discussion

EVAR has advantages over open surgical repair, such as a reduction in anesthesia time, pain and trauma, length of hospital and ICU stay, and blood loss [3], with the potential of experiencing more complications. The patient's history of metastatic lung cancer, known to enhance pro-thrombotic states, likely exacerbated the risk of extensive thrombus formation within the endograft. The thrombus formation could be also associated with the endograft itself, the aneurysm diameter, and the device fabric [4]. The aorto-uni-iliac presentation and polyester fabric were risk factors for IPMT formation [5], as well as a wider main aneurysmal diameter and smaller diameter of limb grafts [6]. Our patient had the aorto-uni-iliac configuration, and a larger aneurysm diameter than the limb grafts, but the type of fabric was unknown. Additionally, her history of metastatic lung cancer gives her a pro-thrombotic status [7]. All these factors could have contributed to the formation of the extensive endograft mural thrombus over the years, as well as potentially contributing to her left renal arterial chronic occlusion and, subsequently, the atrophic/cystic appearance of her left kidney. According to the Food and Drug Administration (FDA) [8] panel, patients should have a post-EVAR 30-day follow-up and lifelong annual surveillance. The Society of Vascular Surgery [9] recommends a surveillance imaging modality with color duplex ultrasound (i.e., no radiation, less cost, and no nephrotoxic agent), contrast-enhanced color duplex ultrasound, or three-dimensional contrast-enhanced ultrasound to detect type I and III endoleaks, and sac enlargement. Further, obtaining CT imaging one year after EVAR and does not show an endoleak and has a stable sac size, or a type II endoleak, it was safe to continue further surveillance with ultrasound. Pandey et al. [10] recommend CT angiography (CTA) as EVAR imaging surveillance with initial tests at 30 days, six months, and one year post procedure and an annual CT if no complications were found in the CTAs. The surveillance strategies could continue changing and improving, and it is important to continue tracking the new recommendations and guidelines to ensure better treatment for this population of patients.

## Conclusions

EVAR with the use of aortic stent-grafts has been an important tool to manage AAA cases without using an open surgical approach. EVAR can have early or late complications at a higher rate than with an open surgical approach. The occlusion of a renal artery in the setting of extensive IPMT in an abdominal aortic endograft is a rare complication of EVAR procedures. Appropriate surveillance and follow-up of these patients are very important to identify the early onset of complications.
